# Carnival or football, is there a real risk for acquiring dengue fever in Brazil during holidays seasons?

**DOI:** 10.1038/srep08462

**Published:** 2015-02-16

**Authors:** Maíra Aguiar, Filipe Rocha, José Eduardo Marques Pessanha, Luis Mateus, Nico Stollenwerk

**Affiliations:** 1 Centro de Matemática e Aplicações Fundamentais, Lisbon University, Portugal; 2 Secretaria Municipal de Saúde de Belo Horizonte, Brazil; 3 Observatório de Saúde Urbana de Belo Horizonte, Minas Gerais Federal University, Brazil; 4 Laboratório de dengue e febre amarela, Fundação Ezequiel Dias, Minas Gerais, Brazil

## Abstract

More than 600,000 football fans, coming from all over the world, were expected to visit Brazil during the FIFA World Cup 2014. International travel can become a public health problem when the visitors start to become sick, needing medical intervention and eventually hospitalization. The occurrence of dengue fever infections in Brazil is persistent and has been increasing since the 1980s, and the health authorities were expected to take preventive measures and to warn the visitors about the risks during the tournament period. Before the World Cup started, studies have been published stating that dengue could be a significant problem in some of the Brazilian cities hosting the games. These conclusions were taken after a brief observation of the available data, analyzing its mean and standard deviation only, or based on seasonal climate forecasts, causing alarm for the world cup in Brazil. Here, with a more careful data analysis, we show that the seasonality of the disease plays a major role in dengue transmission. The density of dengue cases in Brazil is residual during winter in the Southern hemisphere (mid June to mid September) and the fans of football were not likely to get dengue during the tournament period.

Dengue is a viral mosquito-borne infection and a leading cause of illness and death in the tropics and subtropics. It is estimated that 390 million dengue infections occur every year, of which 96 million manifest symptoms with any level of disease severity^2^. In many countries in Asia and South America dengue fever/dengue hemorrhagic fever (DF/DHF) has become a substantial public health concern leading to serious social-economic costs. There is no specific treatment for dengue, and a vaccine which simulates a protective immune response to all four serotypes is not yet available. Tetravalent vaccines are under investigation^3^,^4^, but so far, prevention of exposure remains the only alternative to prevent dengue transmission.

In Brazil, the occurrence of dengue fever is persisting and is increasing since the 1980s. By 2000, dengue virus (DENV) transmission was reported in 22 of the 27 Brazilian states^7^, occupying a significant place in the international ranking for total cases of the disease, according to the World Health Organization (WHO)^5^,^6^. The disease outbreak starts during the rainy season when vector infestation increases considerably, and the suspected cases are of obligatory notification. All reported cases from public health services or private health providers are included in the Sistema de Informação de Agravos de Notificação (SINAN), which is the Brazilian notification database, openly accessible via the internet^8^.

International travel is constantly introducing new vectors and pathogens into novel geographic areas, and could also lead to a local public health problem when the tourists start to become sick during their visit period. Knowing that sports fans and tourists were expected to come from all over the world, frequently with a naive immune system which is not able to protect them against some of the local endemic pathogens that they will probably be exposed to, local authorities are trained to warn the visitors about the risks and were expected to take preventive measures to avoid significant public health problems.

This year's Fédèration Internationale de Football Association (FIFA) World Cup 2014 tournament was held in Brazil, during the winter season on the southern hemisphere, starting on June 12 and ending on July 13. According to estimates from the Brazilian Tourism Ministery, more than 600,000 football fans were visiting the country during the event. The football World Cup games were staged across twelve cities in Brazil: Belo Horizonte, Brasília, Cuiabá, Curitiba, Fortaleza, Manaus, Natal, Porto Alegre, Recife, Rio de Janeiro, Salvador and São Paulo, and the risk of acquiring dengue infection during the tournament period has became of major interest for both, travelers and public health authorities.

In November 2013, an opinion by Simon Hay, published in “Nature”^1^, stated that dengue fever could be a significant problem in Fortaleza, Natal and Salvador. The author claimed that much could be done by the Brazilian authorities to reduce dengue risk in the run-up to the tournament and advised travelers to “select accommodation with screened windows and doors and air conditioning; use insecticides indoors; wear clothing that covers the arms and legs, especially during early morning and late afternoon, when the chance of being bitten by infected mosquitoes is greatest; and apply insect repellent to clothing and exposed skin”. These conclusions were taken after a brief analysis of the mean and the standard deviation for the available data, from 2001 to 2013, of the twelve cities in Brazil that were hosting the games. However, from the information obtained publicly before the event, we observe that there was not a real reason to cause fear for the football World Cup in Brazil, and especially also to promote expensive accommodations where screened windows and doors and air conditioning is required, for example.

More recently, a paper published in The Lancet Infectious Diseases Journal^9^ has predicted high risk of acquiring dengue infection, during the football games in Brazil, for Recife, Fortaleza and Natal cities. These findings were based on seasonal climate forecasts, providing probabilistic forecasts of dengue risk, with risk-level warnings for the twelve host cities where they were scheduled to be hosted. In another recently published paper^10^,^11^, the expected number of cases among foreign tourists during the football World Cup would be of 33 cases, varying from 3–59 cases in 607,051 expected visitors, for all twelve cities during the tournament period. The authors estimated the average per-capita risk and the expected number of dengue cases for each host-city based on reported dengue cases to the Brazilian Ministry of Health solely for the period between 2010–2013, predicting risk only for Fortaleza and Natal, however, lower than it was reported in Ref. 9.

It is important to mention that, up to now, after the event, the publicly available official data refer to dengue notification and dengue confirmed cases from 2001 to 2012 only. The official number of confirmed cases in foreigners during the FIFA World Cup in Brazil is not yet available, however, not more than 3 cases were reported so far, according to Prof. Eduardo Massad (personal communication, September 3, 2014).

It is known that a systematic data collection and its correct analysis should be performed in order to minimize false predictions generated by using wrong data^13^. The above mentioned studies had a big repercussion worldwide, causing alarm among football fans and public health authorities, interfering in the local intervention strategies. But would dengue be effectively a threat during the football tournament? What about Carnival, for example, which is the most famous holiday in Brazil, usually during the summer season, attracting millions of people from all over the world? According to the Brazilian Tourism Ministry^12^, Rio de Janeiro alone received 920,000 tourists during this Carnival in 2014. Bahia (Salvador), Ceará (Fortaleza) and Pernambuco (Recife) together received 1, 6 million tourists, but the number of dengue cases among the visitors has never been a concern.

In this manuscript a more careful data analysis for the Brazilian cities hosting the games is performed. The data was normalized to be analyzed in densities, since travelers meet an average of people while visiting a city, equally if it is a city of e.g. 100,000 people or 5,000,000. Our analysis shows that the risk of acquiring dengue infection in Brazil is seasonal and increases during the rainy season, from mid September till mid May^14^, where the vector infestation increases considerably^17^,^18^. The density of cases becomes residual during the months of June and July. From August to December, cases are rare in all twelve analyzed cities. The risk of being infected by a dengue virus during the tournament period, as well during the Olympic Games that will take place in the city of Rio de Janeiro in 2016, is considered to be lower during winter and spring (from mid June to mid December) than during summer and autumn (mid December to mid June), and therefore the fans of football were not likely to get dengue during the tournament period.

## Methods

In this study, we have analyzed the available epidemiological dengue data for the Brazilian cities which hosted the football games during the FIFA World Cup in 2014. The data were obtained from the Brazilian notification database SINAN^8^, before the event. We analyzed the monthly number of confirmed dengue cases, just as described in Ref. 1. Although the number of confirmed dengue cases from SINAN for 2013 and 2014 were not publicly available, even after the event, the Secretaria Municipal de Saúde^21^ made available the official data of confirmed dengue cases from Belo Horizonte city, to be used as a case study, allowing us to infer how the epidemic patterns of dengue were expected to be observed in the other cities, and to be confirmed when the official number of cases from 2013 and 2014, for the other cities, will become available as well. Since the population size is different for each one of the Brazilian cities, we assume that the disease transmission is density-dependent, since travelers would meet a certain number of locals, independent of the total size of large cities. The population sizes of the Brazilian cities were obtained from the Instituto Brasileiro de Geografia e Estatística (IBGE)^19^.

Studies have predicted a high risk for travelers to acquire dengue fever in the host cities, claiming to use in their analysis the publicly available data from SINAN, up to 2013. However, it is important to say that during that time, the data were not complete after 2012 and would still need to be updated. Later on, these data were not available any more. A note was posted at the SINAN web pages, stating that the Health Ministry in Brazil had detected problems on the integrity of the SINAN database, and therefore, the use of the data for 2013 and 2014 on the database was temporarily suspended, till the appropriate corrections were made. Although this note is not visible anymore, the data for 2013 and 2014 are still not available.

Having said this, we have analyzed for all 12 cities hosting the games, 12 years of publicly available data^8^, except for Belo Horizonte, where 14 years of data were available publicly^20^.

The mean and the standard deviation were analyzed, and the results were compared with the ones published in Ref. 1, in order to show the impact of the population density on the risk of a foreign visitor acquiring dengue infection during the period of the tournament. However, with a highly non-Gaussian and asymmetrical distribution, the mean and standard deviation alone can be difficult to be interpreted. The Gaussian distribution is characterized by the parameters mean and standard deviation and similar distributions can still be understood by these parameters. But the highly asymmetric distributions observed here are much better characterized by e.g. median and some quartiles as used in the so called box-plots.

Moreover, the analyses on the time series via box-plots were performed. The epidemiological data were plotted as time series in order to observe the dynamical behaviour of the dengue epidemics for each one of the Brazilian cities hosting the football games. The data were plotted per 1000 inhabitants per month, since the risk of acquiring dengue infection for a visitor depends on the density rather than the total number of infected in a city.

The conclusions of this study were taken based on the results obtained from the time series analysis via box-plots, combined with the information given by the precipitation data in Brazil, from the Instituto Nacional de Meteorologia (INMET)^22^. By now, we can also conclude that the analysis done for Belo Horizonte using the most complete data set, from 2001 to 2014, has shown the same results with no qualitative changes observed between the analysis using data from 2001 to 2012 and from 2001 to 2014, confirming the prediction we made before the event started.

## Results

The available dengue data for the Brazilian cities which were hosting the football games during the FIFA World Cup in 2014 were obtained in the SINAN Brazilian database. Up to now, after the end of the event, the data which are publicly available are from 2001 to 2012 only, and in order to infer the dengue epidemic pattern for the years os 2013 and 2014 for all cities hosting the games, we use Belo Horizonte city as a case study. It is important to mention that the analysis performed using the updated data, that were obtained after the event and that will be shown in the following sections, has confirmed the prediction we made before the event started, at the time of first submission of this article, using the previously available data.

Belo Horizonte is the capital city of the Brazilian state of Minas Gerais, located in the South Eastern region of the country. As of 2013, the municipality's population is around 2.5 million, making it the most populous city in the State of Minas Gerais and the sixth most populous city in the country. In 2013, the State of Minas Gerais has faced the largest dengue epidemic in its history, with more than 250 thousand confirmed dengue cases, more than 95,000 in Belo Horizonte city alone. For 2014, the number of confirmed cases in Belo Horizonte is at endemic level, expecting less than 1 case per 1000 inhabitants. The official data of confirmed dengue cases in Belo Horizonte for the years of 2013 and 2014 were made available by the Secretaria Municipal de Saúde de Belo Horizonte^21^.

### Time Series Analysis

**Figure 1:**
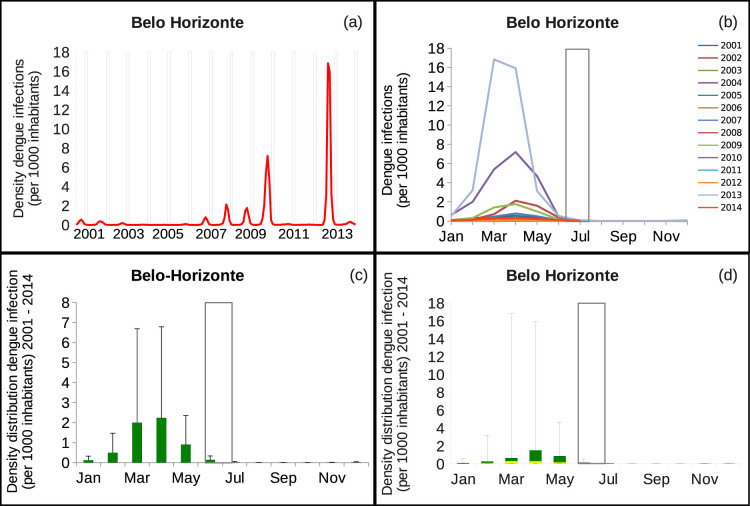
Number of confirmed dengue cases per 1000 inhabitants for the city of Belo Horizonte, from 2001 to October 2014. In (a) time series and in (b) annual time series plot per month, in (c) mean and standard deviation analysis and in (d) analysis via box-plots. Note that for the year 2014, the final 2 months of data need 60 days to be completed. The white bars confine the period where the football World Cup took place, from 12 of June to 13 of July 2014.

We have analyzed the density of confirmed dengue infection in the city of Belo Horizonte. From 2001 to October of 2014 we observed two large outbreaks, in 2010 and 2013. For 2014, the numbers of cases are lower up to now, after the event, (see Fig. 1a), where the white bars confine the period where the Football World Cup was taking place, from 12 of June to 13 of July 2014, confirming the prediction of only a small outbreak to be expected.

In Fig. 1a) and in Fig. 1b) it is possible to see that for all fourteen years of data, the same seasonality pattern is observed, including for the year 2013 (where the largest outbreak has been reported) and for 2014, where the epidemic peak is happening during the months of March and April. Based on this observation, and knowing that the disease is highly seasonal, we assume that for the epidemiological years of 2013 and 2014 the same seasonal behavior will be repeated as it was observed during the past epidemiological years, for each one of the cities that hosted the football games, enabling us to make projections for the risk of being infected during the months of June and July of 2014. In fig. 1c), the mean and standard deviation analysis is shown using densities. Note that the risk of infection is much higher during the summer period (March/April) than during the winter period (June/July). With the analysis via box-plots in Fig. 1d), we observe again, higher risk of infection during the summer, with 0.3 cases per 1000 inhabitants in March and in April, than during the winter, with 0.04 and 0.01 cases per 1000 inhabitants during the months of June and July respectively.

Note that, after the event we are now (in the re-submission phase of this article) able to include the updated data for the city of Belo Horizonte up to October 2014 into our analysis, we confirm the prediction we made before the event started (during the first submission of this study).

**Figure 2:**
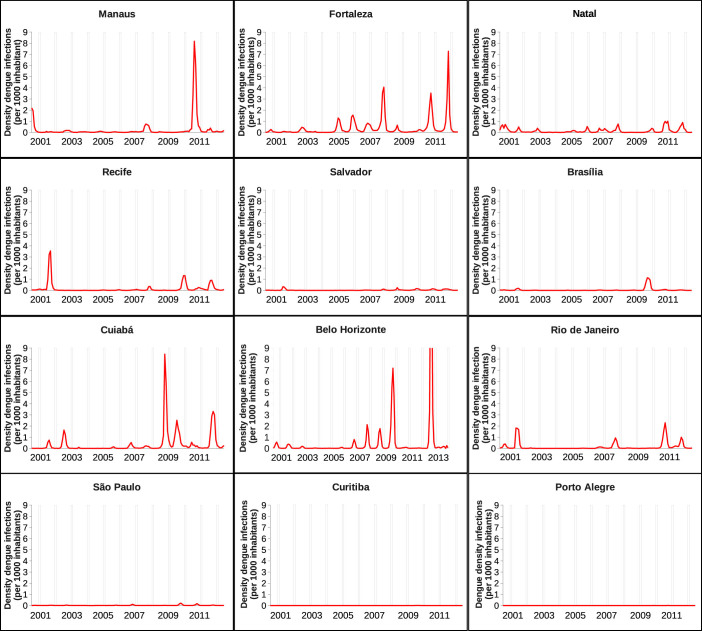
Time series of number of confirmed dengue cases per 1000 inhabitants, from 2001 to 2012, for the Brazilian cities hosting the football games during the FIFA World Cup 2014. Note that the data from Belo Horizonte are now, after the event, updated till October of 2014. The white bars confine the period where the football World Cup took place, from 12 of June to 13 of July 2014.

By looking at the time series panel in Fig. 2 we observe that dengue fever epidemiology dynamics shows large fluctuations of disease incidences in Brazil. Among the twelve selected cities, Fortaleza, Cuiabá and Belo Horizonte appear to be the cities with frequent higher densities of dengue cases in the past six years. Manaus, Recife and Rio de Janeiro have shown a mild density of cases with rarely high outbreaks during the past 12 years. The density of cases is very small in Natal, Salvador, Brasília and São Paulo, and for Curitiba and Porto Alegre, the density of cases is negligible, with only few occasional notified and confirmed cases.

**Figure 3:**
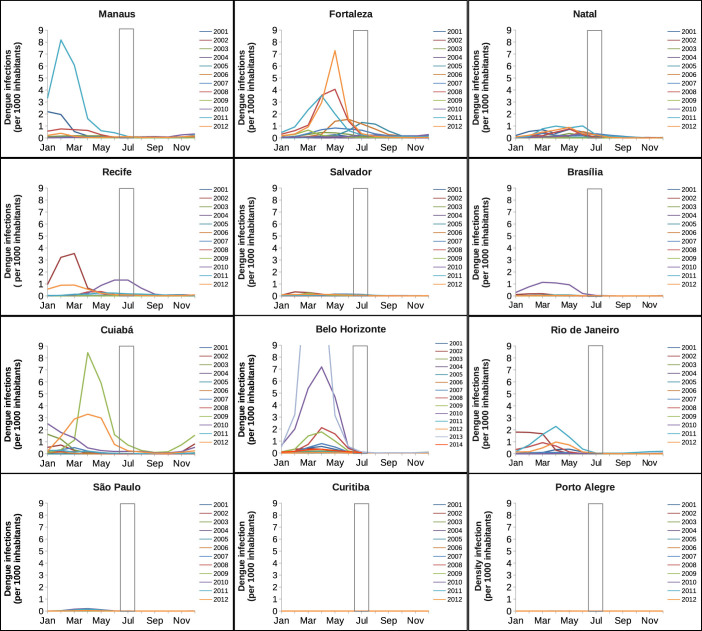
Annual time series of number of confirmed dengue cases per 1000 inhabitants, from 2001 to 2012, for the Brazilian cities hosting the football games during the FIFA World Cup 2014. Note that the data from Belo Horizonte are now, after the event, updated till October of 2014. The white bars confine the period where the football World Cup took place, from 12 of June to 13 of July 2014.

**Figure 4:**
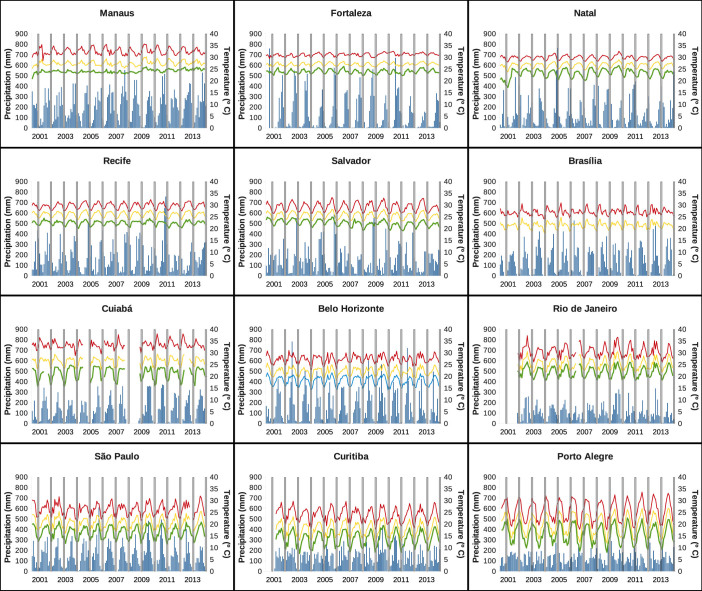
Time series of precipitation and temperature, with temperature given by daily mean, daily maximum and minimum, from 2001 to June 2014, for the twelve Brazilian cities hosting the football games during the FIFA World Cup 2014. The white bars confine the period where the football World Cup took place, from 12 of June to 13 of July 2014. Note that there are missing data for Rio de Janeiro in 2001 and for Curitiba in 2001 and 2008.

In Fig. 3, the twelve years of monthly data, for each one of the cities we are studying, are plotted. We observe that, for all the twelve cities, the dengue season starts in January/February, with the peak of the epidemics around March/April. This is correlated with the increase of vector infestation^17^ and with the rainfall. However, the fluctuations observed in the climatic data shown in Fig. 4 are much smaller than the fluctuations observed in disease incidence. In May, the number of cases starts to decrease considerably, and in June and July, during the football games period (signaled by grey bars in the graphics), the number of cases are residual. This pattern is also confirmed to happen for the other cities that are hosting the football games. Exceptions on the described pattern, where the peak of the dengue epidemic occur during the winter period are observed in Fortaleza in the years of 2005 and 2006 and in Recife in the year of 2010, although with relatively low numbers of infections, less than 2 per 1000 individuals. Note that for Belo Horizonte, our analysis is now done including the recent years of 2013 and 2014, confirming the expected pattern of seasonality of the disease, which we inferred before the event started. Since the official data is not yet available for the other cities, even after the event, we kept the assumption that the epidemiological years of 2013 and 2014 did follow the same behavior observed for each one of the hosting cities, as is possible to observe when looking at the past epidemiological years. We see that there is not a real risk of foreigners being infected, in any of the cities, during the months of June and July of 2014.

**Figure 5:**
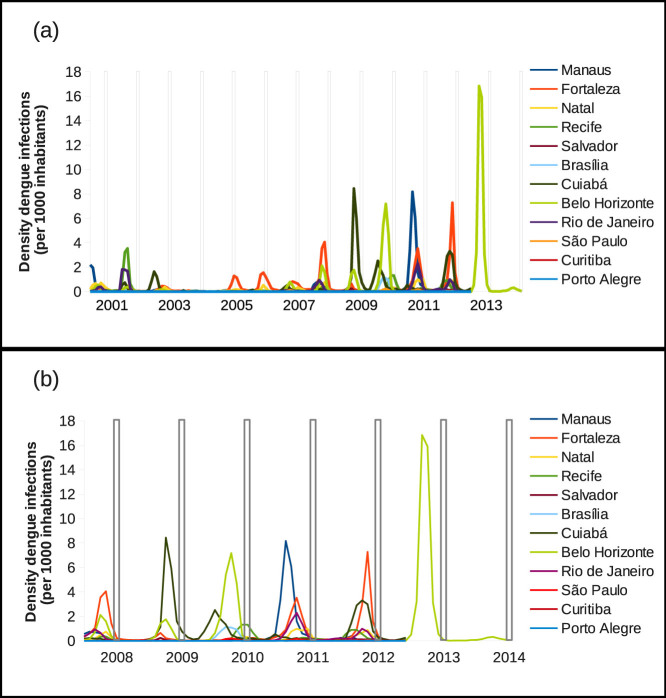
In (a) time-series of the past 12 years for all of the twelve Brazilian cities that hosted the FIFA World Cup in 2014. In (b) time-series of the past 5 years for all of the twelve Brazilian cities that where hosting the FIFA World Cup in 2014. The white bars confine the period where the football World Cup took place, from 12 of June to 13 of July 2014. Note that the data from Belo Horizonte are now, after the event, updated till October of 2014.

From the observation that dengue outbreaks have frequently been large in the past 6 years in Forlaleza, Cuiabá and Belo Horizonte, for example, we can observe that for the years following the high outbreaks, a relatively small outbreak is normally observed. That indicates that the majority of the susceptible individuals are burned out during the high outbreaks, and it takes time to build up the susceptible population for a given dengue strain. Dengue fever epidemiology dynamics shows large fluctuations of disease incidence and the dengue serotype replacement has been observed in empirical data and described with mathematical models of multi-strain interaction^13^,^15^,^16^. In Fig. 5, we can see in more detail that after a high outbreak happened, at least three years are needed before one can see another high outbreak. This pattern is observed for all of the twelve cities, where a smaller outbreak season is always following a large dengue fever outbreak season. This observation allows us to infer that the epidemic year of 2014 will be probably small in cities which have recorded a large outbreak in 2012 (Fortaleza and Cuiabá) and 2013 (Belo Horizonte). Note that, looking at the 2014 data from Belo Horizonte city, the prediction we made before the tournament is confirmed. In 2014, Belo Horizonte reported a very low outbreak, with less than 1 case per 1000 inhabitants (see Fig. 1).

### Mean and Standard Deviation Analysis

In Hay (2013)^1^, an alert of high risk of acquiring dengue infection in the city of Fortaleza, Natal and Salvador during the football games, in June and July of 2014, was communicated to the visitors and to public health authorities, causing fear and eventually interfering with the local control dengue measures, which could have lead to serious economic problems. The conclusions were taken after a brief analysis of the mean and the standard deviation for the available data in absolute numbers, from 2001 to 2013 (even though the data of 2013 was publicly stated to be incomplete and provisory in the SINAN database), for all the twelve cities in Brazil that were hosting the FIFA football games in 2014.

The mean of the confirmed dengue cases for each one of the twelve cities is the average of cases which is computed as the sum of all the dengue cases divided by the number of dengue outbreaks. One problem with using the mean, is that it often does not depict the typical dengue outbreaks. In case of one dengue outbreak that is much larger than the others, the mean will be strongly affected by this single large outbreak.

The standard deviation analysis basically provides an indication of how far the statistical data set varies or deviates from the mean. If the dengue cases all lie close to the mean, then the standard deviation will be small, whereas when the data is spread out over a large range of values, the standard deviation will be large.

**Figure 6:**
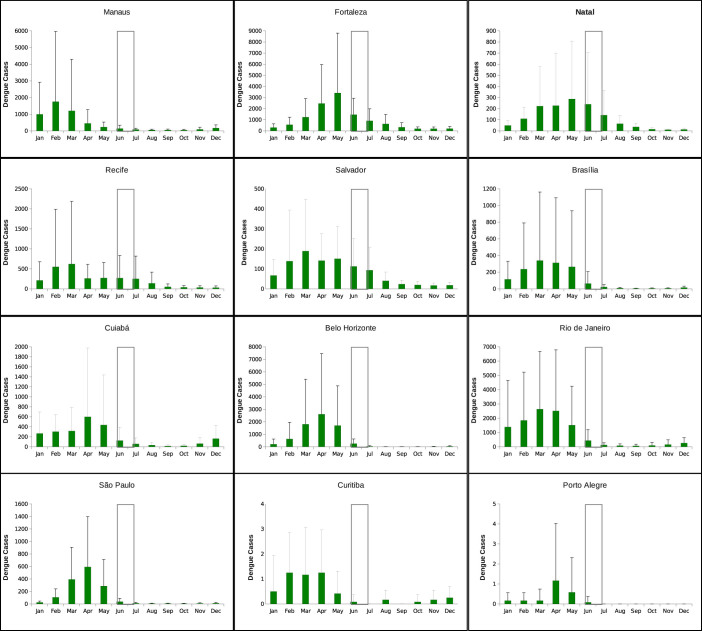
Mean and standard deviation for the twelve Brazilian cities hosting the football games during the FIFA World Cup 2014. Here, the analysis is done using the absolute number of cases, from 2001 to 2012. Note that the time series are plotted in free scale. The data from Belo Horizonte is now, after the event, updated till October of 2014. The white bars confine the period where the football World Cup took place, from 12 of June to 13 of July 2014.

**Figure 7:**
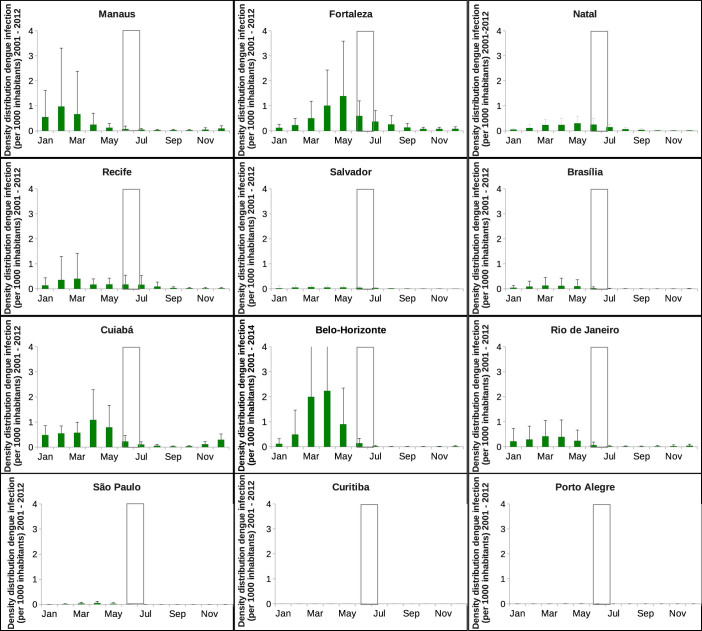
Mean and standard deviation for the twelve Brazilian cities hosting the football games during the FIFA World Cup 2014. Here, the analysis is done using the density data, from 2001 to 2012. Note that the data from Belo Horizonte is now, after the event, updated till October of 2014. The white bars confine the period where the football World Cup took place, from 12 of June to 13 of July 2014.

Fig. 6 shows the mean and standard deviation analysis as it was presented in Ref. 1. The analysis was done using the raw data, and by looking at those graphics, one could superficially conclude that, with exception of Porto Alegre, there is a higher risk of infection during carnival period (Feb./March) than during the FIFA World Cup period (and the next Olympic Games, that will be held in Rio de Janeiro in August 2016). When looking at the period of the year when the FIFA tournament took place (June/July), the risk is higher and basically restricted to the cities of Fortaleza, Natal and Salvador cities, just as stated in Ref. 1. Recife could also be considered as an area of risk, as stated in Ref. 9. However, this analysis did not take the population density for each one of the cities into consideration, and the data were not plotted using the same scale for all cities, giving a false impression of high outbreaks and risk of infection in some of the cities hosting the football games during the winter season. Assuming that the risk of infection is density-dependent, since tourist in a given city only meet an average number of people and surrounding mosquitoes but not all the city population, the mean and the standard deviation analysis is shown in Fig. 7. Except for Salvador, Brasíia, São Paulo, Curitiba and Porto Alegre, where the number of cases per 1000 inhabitants is really small during the whole year, the risk of being infected by a dengue virus is observed to be higher during the carnival season (in Southern hemisphere summer) for example, than during the football World Cup period (in winter). Here, only the city of Fortaleza would show the occurrence of a small number of confirmed dengue cases during the tournament period, however, less than 1 infection per 1000 inhabitants. The cities of Natal, Recife and Cuiabá, with less than 0.5 infections per 1000 inhabitants would represent a very low risk, and for the other cities, including the city of Salvador, the risk of a visitor acquiring dengue infection during the tournament period would be negligible.

**Figure 8:**
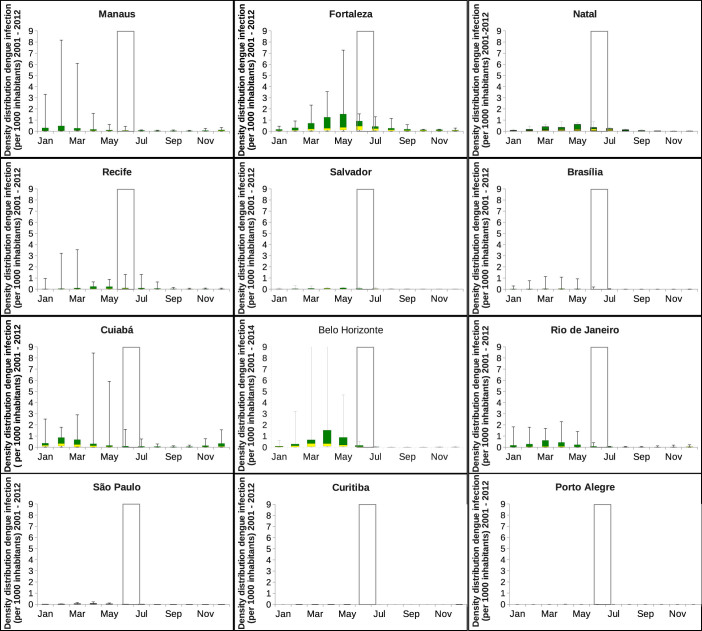
The analysis via box-plots for the density of dengue infection for the Brazilian cities hosting the football games. Note that the data from Belo Horizonte is now, after the event, updated till October of 2014. The white bars confine the period where the football World Cup took place, from 12 of June to 13 of July 2014.

The population density plays a major role in the dengue infection risk estimation. The mean and standard deviation analysis would be well informative when de data set is Gaussian distributed, which is not the case here, as shown in Fig. 2 and in Fig. 8. An alternative measure is the median (50%) and the quartiles, obtained by the analysis via box-plots, where assumptions of the underlying statistical distribution is not needed. The analysis via box-plots is more robust, not giving an arbitrarily large result when describing the typical value, and especially informative when the distributions are highly asymmetric.

### The Analysis Via Box-plots

Using the box-plots to characterize the distribution of cases in each city hosting the football games during the FIFA World Cup 2014, we present the minimum value, the 25% percentile (first quartile in yellow), the median, the 75% percentile (third quartile in green) and the maximum value of density distribution of the dengue infection per 1000 inhabitants, for the twelve Brazilian cities that were hosting the FIFA World Cup football games. The data are highly non-Gaussian distributed and the analysis via the box-plots (see Fig. 8) gives a more realistic portrait of the distribution of dengue epidemics in Brazil and a better overview on the risk of being infected in a certain city during a given period of the year, as opposed to the mean and standard deviation analysis (see section ‘Mean and Standard Deviation Analysis'). Note that Gaussian distributions only give symmetric box-plots, with mean and median equal, where non-Gaussian distribution have in general a different median from the mean and asymmetric quartiles.

Cities with frequent high outbreaks appear with a large median and large third quartile, while cities with frequent lower outbreaks appear with a small median. In Fig. 8, we observe that only the city of Fortaleza is reporting the highest outbreaks, less than 0.5 and around 0.2 cases in June and in July respectively, per 1000 inhabitants. Natal, with approximately 0.2 per 1000 inhabitants, would represent a small risk of infection, and for the other cities, the risk is close to zero, during the tournament period. For the carnival season, low risk of infection is also expected, with not more than 0.4 cases per 1000 inhabitants in Cuiabá, around 0.2 in Fortaleza, Natal and Belo Horizonte, enabling us to assume that the public heath interventions before and during the summer/autumn seasons are effective and to some extend efficient in controlling the disease transmission.

## Discussion

Holiday seasons and big sports events such as the FIFA World Cup and the Olympic Games bring thousands and millions of people to the host country. Although it is considerably good for the local economy, international travel constantly introduces new vectors and pathogens into novel geographic areas, and could also lead to a local public health problem.

The FIFA World Cup 2014 took place in Brazil and more that 600,000 foreign visitors were expected. Brazil is a tropical country, and dengue fever is one of the major public health concerns. Many publications have come out recently alerting the football fans about the risks of acquiring dengue infection in Brazil, during the football games, which took place from 12 of June to 13 of July, during the local winter season. An opinion published in “Nature”^1^, based on the mean and standard deviation analysis only, concluded that there was a high risk of being infected by a dengue virus in Fortaleza, Natal and Salvador, and football fans should be especially attentive when visiting those cities. More recently, a paper published in The Lancet Infectious Diseases Journal^9^ conclude based on climatic forecasts, that the risk of being infected in Brazil during the tournament would be high in Recife, Fortaleza and Natal. In Refs. 10, 11, an average of 33 cases were expected and the risk was stated to be in Recife and in Fortaleza only, however, on a smaller scale than reported in Ref. 9.

In this publication, a more careful data analysis of the dengue fever data for the Brazilian cities that were hosting the games was performed, and we have shown that the risk of being infected by a dengue virus is seasonal, increasing during the rainy season, and the presence of vector and human population density. We have analyzed the epidemiological dengue data, where confirmed cases from 2001 to 2012 were taken into consideration for the twelve Brazilian cities that were hosting the football games during the FIFA World Cup 2014. The city of Belo Horizonte was taken as a case study using the information from 2013 and 2014 also, and the epidemic pattern of the disease was analyzed and the results were extrapolated to the other cities, with attention to the peculiar seasonal pattern observed in the previous years, for each city. From that, we assumed that, for each host cities, the pattern would follow the same dynamics in 2013 and in 2014 as was observed, for a given city, in the previous years, from 2001 to 2012. After the event, the updated data from Belo Horizonte, from 2001 to October 2014, were included in the analysis, confirming the predictions given before the event.

We have observed that dengue epidemics in Brazil is highly seasonal, with its peak mainly during the months of March and April. Since the risk of a foreign visitor acquiring dengue infection is density dependent, we analyzed the epidemiological data by taking into consideration the population density for each of the twelve Brazilian cities. Our results based on the analysis via the box-plots have shown that during the Brazilian winter season (from mid June to Mid September), when the FIFA World Cup has happen, the only city that could represent a (low) risk for foreign visitors is Fortaleza, with approximately 0.4 case per 1000 inhabitants observed during the month of June and 0.2 case per 1000 inhabitants observed during July. For Natal, the risk is even lower, with less than 0.2 cases per 1000 inhabitants during the months of June and July. For the other cities, the risk of acquiring dengue during the months of June and July, including Recife, would be close to zero, and hence considered negligible.

Our analysis has also shown that during the summer and the autumn seasons, when carnival (or summer holidays, for example) usually take place, attracting millions of people from all over the world, the risk of being infected by a dengue virus in Brazil exists in a very low scale. With no more than 0.4 cases per 1000 inhabitants in Cuiabá and around 0.2 in Fortaleza, Natal and Belo Horizonte, for example, we find these areas to be of higher risk of dengue infection as compared to other cities that hosted the games. However, the existing risks for such a season are still kept quite low, enabling us to assume that the public heath interventions before and during the summer/autumn seasons are effective and to some extend efficient in controlling the disease transmission.

It is important to emphasize though that the risk increases when a high epidemic year is expected to happen. Based on the interpretation of the box-plots, the median of cases during summer is not much higher than the risk predicted during the winter season in Brazil, in average varying from 0.2 to 0.5 per 1000 inhabitants. As for the upcoming event in Brazil in 2016, the Olympic Games, that will take place in Rio de Janeiro, the risk of being infected by a dengue virus is expected to be negligible.

Those findings have important implications for the effectiveness of intervention measures that will be provided by the Public Health Authorities for dengue control in Brazil, as well for the economic impact that a wrong prediction of risk of infection could cause. The correct data interpretation and its appropriate analysis is of major importance, able to minimize false predictions of risk of infection. However, up to now, after the end of the tournament period, there are no publicly available data for the confirmed dengue cases in Brazil for 2013 and for 2014 to evaluate the risk a posteriori.

## Author Contributions

M.A. and N.S. designed and coordinated the study; M.A., N.S. and F.R. performed the data analysis; M.A., F.R., J.E.M.P., L.M. and N.S. wrote the paper.
